# Monocyte Chemoattractant Protein-1 in Antineutrophil Cytoplasmic Autoantibody-Associated Vasculitis: Biomarker Potential and Association with Polymorphisms in the MCP-1 and the CC Chemokine Receptor-2 Gene

**DOI:** 10.1155/2018/6861257

**Published:** 2018-03-12

**Authors:** Nina Jönsson, Evelina Erlandsson, Lena Gunnarsson, Åsa Pettersson, Sophie Ohlsson

**Affiliations:** Department of Nephrology, Institution of Clinical Sciences in Lund, Lund University, Lund, Sweden

## Abstract

Antineutrophil cytoplasmic autoantibody- (ANCA-) associated vasculitis (AAV) are relapsing-remitting disorders with unpredictable prognosis. There is a need of biomarkers for distinguishing which patients will have a more severe outcome and also for predicting relapses in disease activity. This study confirms the previous results of urinary MCP-1 (uMCP-1) as a prognostic marker and explores its potential as a marker of disease activity. *Method*. 114 patients with AAV were followed regularly between 2002 and 2011 at Skåne University Hospital. Urine samples, blood samples, and clinical status were registered. The urine samples were analyzed in an in-house-developed ELISA. PCR-RLFP was used to analyze the MCP-1 and CCR2 genes. *Results*. Patients with severe prognosis had significantly higher levels of uMCP-1 compared to patients with nonsevere prognosis and healthy controls. Patients with renal damage had higher levels compared to patients who did not have renal damage. There was also a tendency of higher uMCP-1 levels in active disease as compared to remission. AA in the -2518 position in the MCP-1 gene was associated with a more severe outcome compared to the A/G or the G/G genotype. The A/A genotype were also associated with higher levels of uMCP-1. No significant associations were seen for the CCR2-V64I. *Conclusion*. This study confirmed the connection between high uMCP-1 levels and poor prognosis and also disease activity. It also suggests an association of the A/A genotype at position -2518 in the MCP-1 gene and poor prognosis in AAV. uMCP-1 is clearly a candidate biomarker of potential clinical value. The A/A genotype association needs further evaluation.

## 1. Introduction

The antineutrophil cytoplasmic autoantibodies (ANCA) were discovered in 1982 in 8 patients with segmental necrotizing glomerulonephritis [1]. Major targets for ANCA are myeloperoxidase (MPO) and proteinase 3 (PR3) in monocytes and neutrophils [2]. ANCA is strongly associated with three types of small vessel vasculitis: microscopic polyangiitis (MPA), granulomatosis with polyangiitis (GPA), and eosinophil granulomatosis with polyangiitis (EGPA) [3].

Most patients with GPA have ANCA against PR3 and most patients with MPA have ANCA against MPO, whereas in EGPA, the majority is ANCA negative although MPO-ANCA occur. In systemic GPA, ANCA positivity is seen in more than 90% [3, 4]. About 10% of patients with GPA or MPA have negative assay for ANCA, especially those with less severe disease [5, 6].

ANCA is capable of activating the leukocytes to adhere to the endothelial cells in the vessel wall and of inducing an inflammatory process. It is thereby thought to play a role in the pathogenesis of small vessel vasculitis [7, 8]. Patients with ANCA against PR3 have been shown to have more frequent respiratory tract granulomas and extra renal organ manifestations compared to patients with ANCA against MPO. Anti-PR3 ANCA has also been seen to correlate with a faster decline in renal function and more frequent relapses compared to anti-MPO-ANCA [9].

ANCA-associated vasculitis affects mainly capillaries, and also arterioles and venules, and in some cases middle-sized blood vessels. AAV is most common in adults in their '50s and '60s. The distribution among men and women is equal [5]. Prevalence in the healthcare district around the city of Lund in southern Sweden per million inhabitants is 94 for GPA, 31 for MPA, and 14 for EGPA, which is the highest prevalence reported in the world so far [10]. The 5-year survival for GPA is 74–91%, for MPA 45–76%, and for EGPA 60–97% [11].

Cyclophosphamide and glucocorticoids have been used as treatment in AAV for over 40 years, and the combination is the standard treatment for remission induction. If the patient has normal renal function and a less severe disease, a combination of methotrexate and glucocorticoids can be used instead as a less toxic option. To maintain remission, low doses of glucocorticoid and either methotrexate, azathioprine, or sometimes mycophenolate mofetil are recommended. Other treatment such as immunoglobulin or rituximab can be considered as alternative treatment in relapsing, refractory, or persistent disease [12]. Treatment with cyclophosphamide is associated with morbidity and mortality due to myelosuppression, infection, and malignancy [13]. Mortality due to ANCA-associated vasculitis and immunosuppressive treatment has however decreased over the last 4 decades, as dose regimens have been optimized [14].

To further optimize and individualize treatment, there is a need for biomarkers that can predict poor outcome. Renal function at diagnosis is a strong predictor of patient survival [15–17]. Other factors shown to correlate with poor prognosis are IL-8 [18], IgM [19], and high levels of PR3-ANCA measured by capture ELISA [20]. In a previous study, uMCP-1 was shown to be able to predict poor prognosis and the association was stronger not only compared to BVAS, CRP, and ANCA, which are markers used today to follow disease activity, but also compared to new markers shown to be able to predict poor prognosis such as urinary IgM and IL-8 [21].

There is also a need for biomarkers that are able to predict relapse. ANCA levels have been shown to predict relapses in GPA, but the relapses often occur more than 6 months after a rise in ANCA levels and the sensitivity and specificity are not very high [22]. A proteomics study from 2009 describes a number of biomarkers able to distinguish between patients with active AAV and patients in remission. Proteolytic products of hemoglobin were the most frequently observed, but fragments of albumin and *α*1-antitrypsine were also seen [23].

MCP-1, also called chemokine (C-C motif) ligand 2 (CCL2), was the first CC chemokine to be discovered. It is composed of 76 amino acids and the gene is located on chromosome 17. MCP-1 is produced by endothelial, epithelial, smooth muscle, mesangial, astrocytic, monocyte, and microglial cells as well as fibroblasts. Monocytes/macrophages are the major source, and MCP-1 is a potent chemotactic factor for monocytes. uMCP-1 has been shown to be useful as a marker in diseases such as systemic lupus erythematosus (SLE) and diabetes mellitus [24, 25].

Single-nucleotide polymorphism (SNP) in the gene regulatory region at position -2518 A/G in the MCP-1 gene have been reported. Healthy individuals with the A/G or G/G genotype are producing more MCP-1 than individuals homozygous for the A/A genotype [26]. However, another study showed that the A/A genotype was associated with renal disease in SLE and elevated levels of MCP-1 in both serum and urine [27]. The GA and AA genotype is associated with clinical severity in Behcet's disease [28]. The GG genotype is associated with higher risk of chronic obstructive pulmonary disease (COPD) [29], oral squamous cell carcinoma [30], and late acute rejection in kidney-transplanted patients [31].

CCR2 is mainly expressed on monocytes and is the receptor not only for MCP-1 but also for MCP-2, MCP-3, and MCP-4. It has 374 amino acids and the aminoterminal domain is necessary for binding of MCP-1 [32]. In CCR2 polymorphism at position 190 from G to A, a mutation called CCR2-V64I causes a change from valine to isoleucine at codon 64, and it has been shown that this change causes a higher expression of CCR2A compared to CCR2B, which in turn downregulates the cell surface expression of CCR5 [33].

It has been shown that this polymorphism is protective in the development and progression not only of inflammatory diseases like multiple sclerosis (MS) [34] and acquired immunodeficiency syndrome (AIDS) [35] but also of malignancies such as breast cancer [36].

The aim of this study was to further establish the correlation of uMCP-1 levels and poor prognosis shown previously [21]. We also wanted to explore the connection between uMCP-1 levels and disease activity. In addition, the -2518 A/G polymorphism in the MCP-1 gene and the CCR2-V64I polymorphism in the CCR2 gene has been studied.

## 2. Materials and Methods

### 2.1. Patients

Patients who visited the Department of Nephrology at Skåne University Hospital between 2002 and 2010 with an ANCA-associated vasculitis (AAV) diagnosed according to the Chapel Hill criteria were asked to participate in the study. Classification of AAV was done according to the consensus methodology described by Watts et al. in 2007 [37]. Patients on dialysis and patients with cancer were not included. The patients were followed by regular visits at the open patient clinic between 2002 and 2011. Each time, blood and urine samples were taken and clinical status was registered. Clinical status was measured using the Birmingham vasculitis activity score (BVAS) [38] and development of critical damage was according to the vasculitis damage index (VDI) [39]. Clinical status was classified as remission (BVAS 0-1), chronic grumbling activity (BVAS 2–5), or relapse/new disease activity (BVAS > 6). The patient was considered having a severe prognosis if the patient fulfilled at least one of the following criteria: (1) chronic kidney failure with glomerular filtration rate (GFR) < 30, (2) stroke after diagnosis, (3) myocardial infarction after diagnosis, (4) subglottic stenosis, (5) respiratory insufficiency requiring oxygen treatment, (6) start of dialysis treatment or kidney transplantation after diagnosis, and (7) death. Patients who did not meet any of these criteria were classified as having a nonsevere prognosis. The control group consisted of healthy blood donors. Urine samples were also collected from 9 patients with other vasculitis diagnoses. All patients and controls gave their written informed consent to participate, and the study was accomplished with permission from the local ethical committee of Lund (see Table 1 for more patient characteristics).

### 2.2. Blood and Urine Samples

Urine samples of first-voided urine were collected in polyethylene vessels (Kebo AB, Sweden). The urine samples were kept frozen at −20°C until assayed. Blood samples were collected in EDTA tubes and centrifuged, and the plasma was stored in −20°C.

### 2.3. MCP-1 Enzyme-Linked Immunosorbent Assay (ELISA)

Human monocytes were isolated from healthy donors using OptiPrep as described by the manufacturer. From the monocytes, total cellular mRNA was isolated using Qiagen mRNA purification kit as described by the manufacturer. cDNA was obtained by one-step reverse transcription polymerase chain reaction (RT-PCR), using a forward oligonucleotide primer (primer D04 F: GAAACTATTTTATCAAAAGCATGC) and a reverse oligonucleotide primer (primer D05 R: GGCAATTATCATAGCCAGCAG). RNAse inhibitor, one-step enzyme reverse transcriptase and deoxyribonucleotide triphosphates (dNTPs) were used during the PCR. The PCR product from the PCR was analyzed on a 1% agarose gel, and MCP-1 (300 bp) was extracted by using MinElute Gel Extraction Kit (Qiagen) as described by the manufacturer. cDNA was cloned into a pCR2.1TOPO (TA 3.9 kb) vector using (TA Cloning Kit, Invitrogen) as described by the manufacturer.

Expression and purification of MCP-1 was performed as previously described [40]. Instead of insect cells, human embryonic kidney (HEK) 293 cells were used. These were cultured in DMEM with 10% fetal calf serum (FCS). Transfection with the plasmid containing the MCP-1 gene was made by electroporation. Selection of the transfected cells was performed using geneticin. Recombinant MCP-1 was purified in two steps. First, the supernatant of the collected serum-free cell medium was added directly on a Mono S HR 55 column. The bound protein was eluted with a 50 min linear gradient from 0 to 1 M NaCl in 20 mM MOPS, pH 6.5. Fractions were collected and to verify MCP-1 content, a capture ELISA using rabbit anti-MCP-1 (Abcam) was used. The fractions containing MCP-1 were assayed in a Western blot to explore the purity using rabbit anti-MCP-1 (Abcam). Fractions containing MCP-1 were pooled. To further purify MCP-1, the fraction was gel filtered on a Superdex 75 HR 1030 column in 0.1 M ammonium acetate. Again, a Western blot and a capture ELISA were performed to find the fractions containing MCP-1, which were pooled. The collected MCP-1 was sent to Innovagen for immunization of rabbits resulting in rabbit anti-MCP-1. Innovagen also biotinylated some of the rabbit anti-MCP-1.

**Figure 1:**
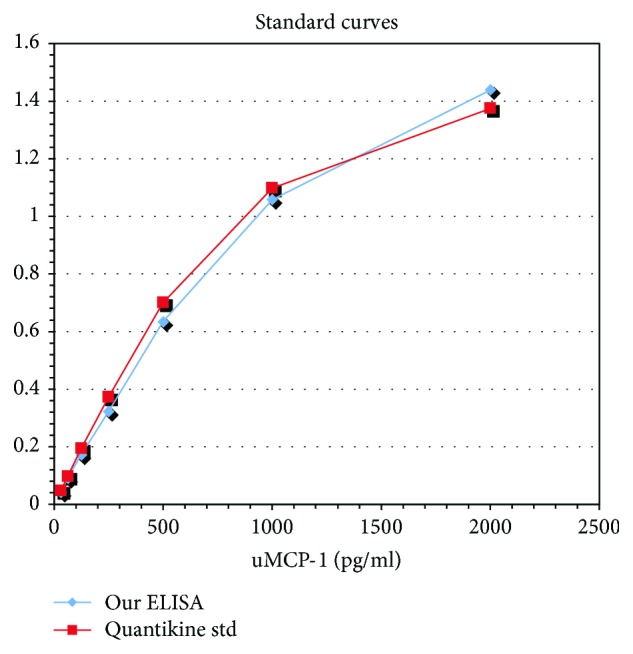
Comparison of the in-house-developed ELISA and the commercial ELISA kit. The graph shows the comparison of ELISA's ability to measure determined levels of added MCP-1, the standard curve in the ELISA. Absorbance at 405 nm, background absorbance subtracted. ELISA: enzyme-linked immunosorbent assay; uMCP-1: urinary monocyte-chemoattractant protein-1.

The ELISA was developed analyzing urine samples with both Quantikine and our own antibodies from Innovagen. The standard curves were similar (see Figure 1). The results from the in-house-developed ELISA had a mean variation of 9.0% compared to the Quantikine kit. A recovery test was made adding 10, 100, and 200 pg/ml Quantikine MCP-1 to four test urine samples with a mean variation of 12.9%. Inter- and intraassay tests were made with a mean variation of 6 and 10%.

Microplates were precoated with rabbit anti-MCP-1 and left in +8°C overnight. The urine samples were diluted 1 : 2 in Tris buffer (50 mM Tris HCl, 0.05% NaN_3_, 0.2% bovine serum albumin (BSA), and 0.05% Tween 20) and then added to the microplates and left to incubate for two hours in room temperature. Biotinylated rabbit anti-MCP-1 was used as a secondary antibody and left to incubate for two hours in room temperature. After that, streptavidin-AP was added to each well and left to incubate for one hour in room temperature. A substrate was added and the absorbance was read after 45 min incubation in room temperature. Between every step, the microplates were washed three times in washing solution (0.9% NaCl, 0.05% Tween).

### 2.4. Laboratory Work-Up

Urine samples were sent to the Clinical Chemistry Department at Skåne University Hospital for measurement of the creatinine levels and urine albumin/creatinine index. Blood samples were sent for analysis of C-reactive protein (CRP), white blood cell count, creatinine, and cystatin C. PR3-ANCA and MPO-ANCA were analyzed at Wieslab, Euro Diagnostica, Malmö.

### 2.5. MCP-1 Polymorphism

The MCP-1 -2518 A/G polymorphism was genotyped using restriction fragment length polymorphism polymerase chain reaction (PCR-RLFP) as previously described [29]. Genomic DNA was extracted from peripheral blood by using AllPrep DNA/RNA Mini Kit (Qiagen) according to the manufacturer's protocol. 0.5 *μ*l forward primer 5′-TCTCTCACGCCAGCACTGACC-3′ and 0.5 *μ*l reverse primer 5′-GAGTGTTCACATAGGCTTCTG-3′ (Invitrogen) were added to the DNA together with 6 *μ*l MgCl_2_, 1 *μ*l dNTPs, 2 *μ*l Taq polymerase, H_2_O, and 5 *μ*l buffer to a total volume of 50 *μ*l. Amplification occurred by 5 min denaturation in 94°C and thereafter 30 cycles of denaturation for 1 min in 94°C, annealing for 1 min in 55°C, and extension for 1 min 30 s in 72°C. The process ended with 7 min extension in 72°C. The PCR products were digested using Pull (Fermenta) at 37°C overnight. MCP-1 -2518 G/A variants were detected by electrophoresis on 3% agarose gel.

### 2.6. CCR2 Polymorphism

Genomic DNA was extracted using AllPrep DNA/RNA Mini Kit (Qiagen). Forward primer 5′-TTGTGGGCAACATGATGG-3′, reverse primer 5′-CTGTGAATAATTTGCACATTGC-3′ (Invitrogen), MgCl_2_, dNTPs, Taq polymerase, H_2_0, and buffer were added to the DNA. The DNA was amplified by denaturation for 5 min in 94°C, 30 cycles of 1 min denaturation in 94°C, 1 min annealing in 57°C and 30 s extension in 72°C, and finally extension for 7 min in 72°C. PCR products were digested with BseJL (Fermenta) for 4 h at 65°C. CCR2-V64I variants were detected by electrophoreses on 3% agarose gel.

### 2.7. Statistics

Statistics and calculations were performed in SPSS version 20. Statistical significance was considered if *p* < 0.05. Analyses of the connection between uMCP-1 and other parameters were performed using the nonparametric tests Kruskal-Wallis and Mann–Whitney *U* test. Correlation of uMCP-1 with other parameters was studied using Spearman's rho test. The polymorphism frequencies were studied by the Pearson chi-square test.

## 3. Results

### 3.1. uMCP-1

**Figure 2:**
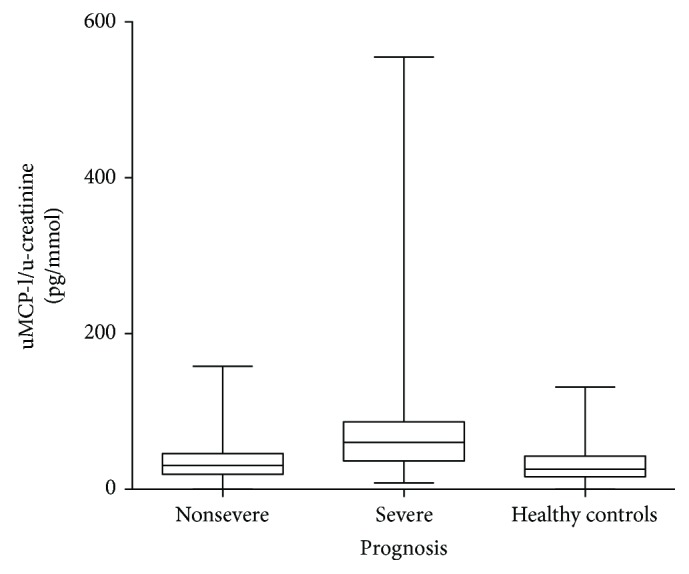
Comparison of uMCP-1 levels in patients with severe prognosis. Nonsevere prognosis and healthy controls. Only urine samples in remission are included. All samples are divided with urinary creatinine levels. uMCP-1: urinary monocyte chemoattractant protein-1. Patients with severe prognosis had higher uMCP-1 levels (*p* < 0.001).

All MCP-1 values were divided by u-creatinine before statistical analysis, in order to compensate for the impact of varying urine concentration. Comparison of the mean MCP-1 levels in remission in urine in patients with severe prognosis, nonsevere prognosis, and healthy controls showed significantly higher uMCP-1 values in patients with severe prognosis (*n* = 46) compared to patients with nonsevere prognosis (*n* = 68, *p* < 0.001) and compared to healthy controls (*n* = 24, *p* < 0.001) (see Figure 2). The patients with nonsevere prognosis did not have significantly higher levels of MCP-1 in urine compared to healthy controls. Patients with other vasculitis diagnoses (*n* = 9) had lower levels than the AAV patients with poor prognosis, but higher than the healthy controls (data not shown).

**Figure 3:**
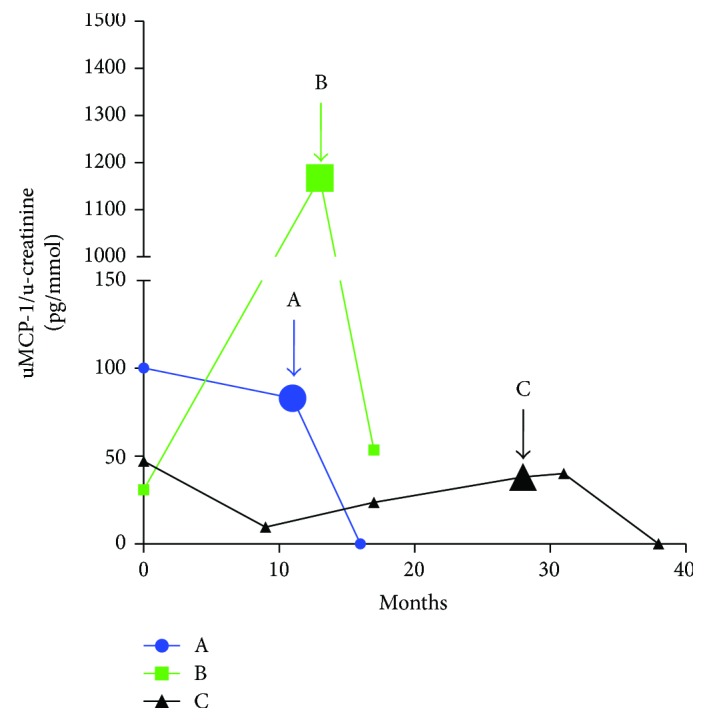
Patients who develop renal damage during the study. Longitudinal view of how uMCP-1 levels changes over time in patients who developed renal damage according to the vasculitis damage index, patients A–C. The *y*-axis was cut to fit in the high levels of patient B. The arrows point at the first sample taken after renal damage was discovered. Only samples in remission are included. uMCP-1 levels are divided with urinary creatinine levels. uMCP-1: urinary monocyte chemoattractant protein-1.

**Figure 4:**
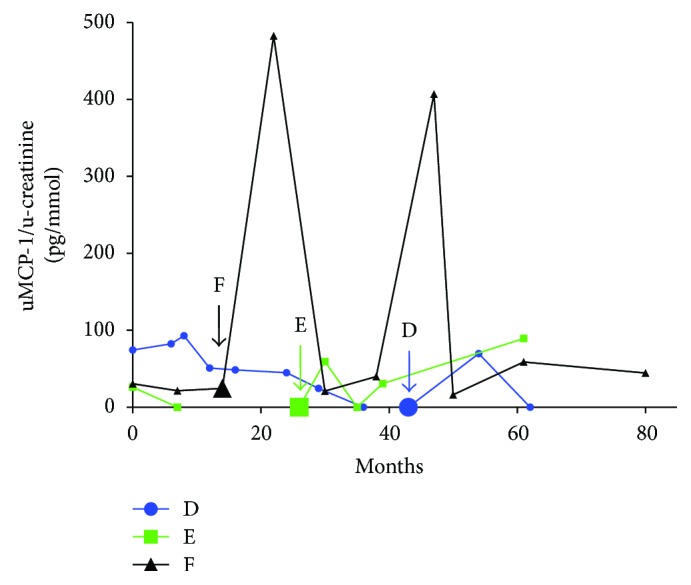
Patients who develop renal damage during the study. Longitudinal view of how uMCP-1 levels changes over time in patients who developed renal damage according to the vasculitis damage index, patients D–F. The *y*-axis was cut to fit in the high levels of patient B. The arrows point at the first sample taken after renal damage was discovered. Only samples in remission are included. uMCP-1 levels are divided with urinary creatinine levels. uMCP-1: urinary monocyte chemoattractant protein-1.

There were 45 patients who scored for renal damage in VDI. Six of these had urine samples taken from both before and after the appearance of kidney damage (see Figures 3 and 4). uMCP-1 seem to peak around the time point of kidney damage. When comparing the mean value in each patient of all the samples taken without/before kidney damage and the mean value of all samples taken with/after kidney damage appeared, uMCP-1 levels were significantly higher when kidney damage had appeared (*p* < 0.0001).

**Figure 5:**
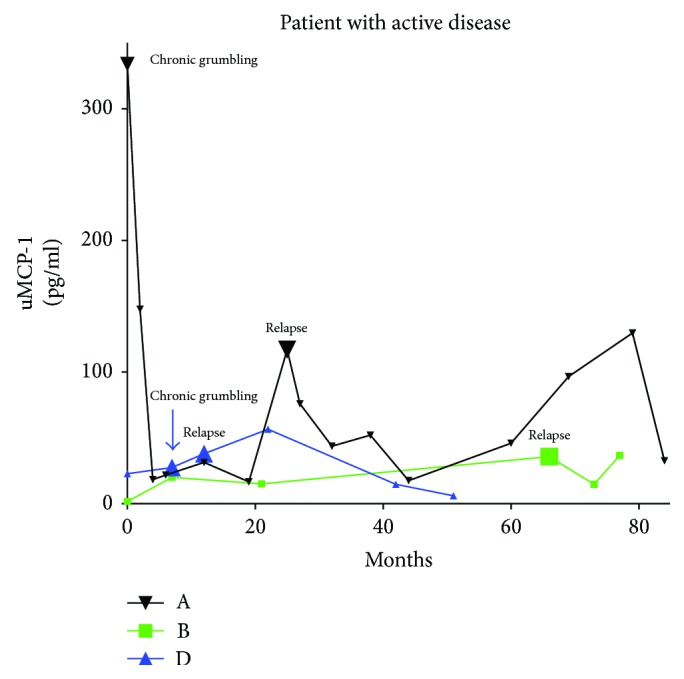
uMCP-1 levels in three of the patients who relapsed during the follow-up. All samples are taken in remission except those labelled relapse in the diagram. uMCP-1 levels are divided with urinary creatinine levels. uMCP-1: urinary monocyte chemoattractant protein-1.

**Figure 6:**
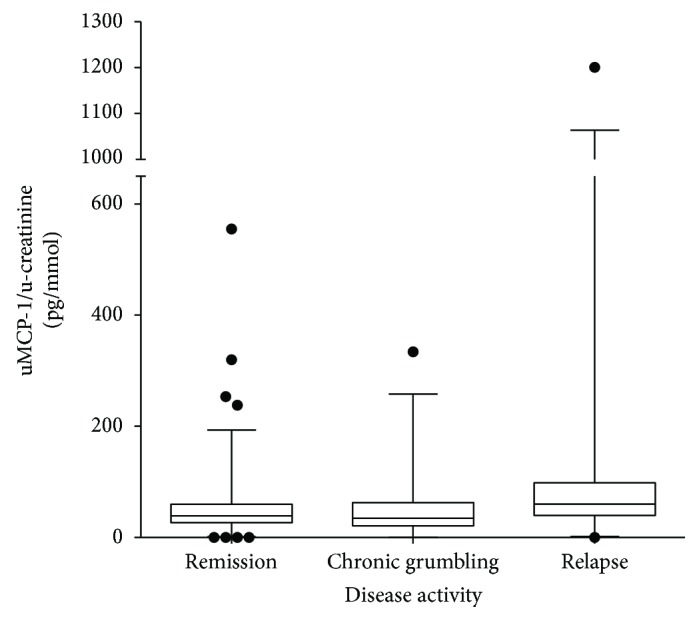
Comparison of uMCP-1 levels in different disease activity. uMCP-1 levels are divided with urinary Creatinine levels. The *y*-axis was cut to fit in the high levels of the relapse group. uMCP-1 levels are divided with urinary creatinine levels. uMCP-1: urinary monocyte chemoattractant protein-1. uMCP-1 was higher in relapse than in remission (*p* < 0.05).

There were 10 patients who had urine samples taken both when in remission and when in the active phase, as demonstrated in Figure 5. Overall, in urine samples taken from patients in remission, the amount of MCP-1was significantly lower than in urine samples taken from patients in the active phase (*p* = 0.023). Urine samples taken when the patients were in the chronic grumbling phase had neither significantly higher levels of MCP-1 compared to samples taken in remission nor significantly lower levels than patients in the active phase (Figure 6).

There were no significant differences in the amounts of MCP-1 regarding diagnosis or gender. Patients with seronegative AAV had significantly lower uMCP-1 levels compared to patients with MPO-ANCA (*p* = 0.046), and no other significant differences were seen regarding ANCA specificity.

Based on the levels of uMCP-1 over the 3rd quartile in healthy controls, the patients' mean uMCP-1 values were classified as high. Levels of uMCP-1 under the 1st quartile in healthy controls were classified as low. When looking at poor prognosis, the positive predictive value was 70% and the negative predictive value was 87.5%.

There was a significant correlation between levels of uMCP-1 and levels of CRP, white blood cell count, and creatinine in serum. There was also a significant correlation with U-albumin/creatinine index (*p* < 0.01) (see Table 2).

### 3.2. Polymorphism in the MCP-1 Gene and CCR2 Gene

**Figure 7:**
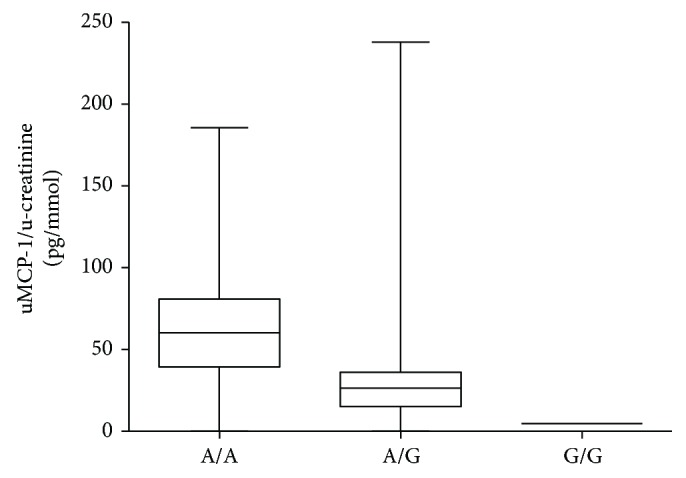
Comparison of uMCP-1 levels in patients with different genotype in the MCP-1 gene at position -2518. uMCP-1 levels are divided with urinary creatinine. uMCP-1 levels are divided with urinary creatinine levels. uMCP-1: urinary monocyte chemoattractant protein-1.

There were no significant differences between the number of patients having the different genotypes at position -2518 in the MCP-1 gene, as compared to the distribution among the healthy controls (see Figure 7 for more details regarding the polymorphism analysis). Neither was there any difference regarding the CCR2-V64I mutation in patients compared to healthy controls. No differences in the investigated genotypes were seen regarding diagnosis or ANCA specificity. However, in patients with severe prognosis, 87.5% had the A/A genotype, 12.5% had the A/G genotype, and 0% had the G/G genotype. This was a significant difference compared to patients with nonsevere prognosis, where 39.5% had the A/A genotype, 59.3% had the A/G genotype, and 5.2% had the G/G genotype (*p* = 0.0068). All patients who developed end-stage renal disease had the A/A genotype. The mean uMCP-1 level was significantly higher in the patients with the A/A genotype compared to the A/G and the G/G genotype (*p* = 0.02) (see Table 3). There was no significant difference in the mean uMCP-1 value in patients when comparing the A/A, A/G, and G/G genotype of the CCR2 gene.

## 4. Discussion

In a previous study [21], it was shown that uMCP-1 could be a potential marker of severe prognosis and maybe also a marker of active disease. In this longitudinal study, we wanted to further explore the ability of uMCP-1 to predict active disease and relapses and also to confirm its role as a prognostic marker. In addition, we wanted to explore which role earlier described mutations in the MCP-1 gene and the CCR2 have in AAV.

Our results showed that patients with a more severe prognosis had significantly higher levels of uMCP-1 compared to patients with a better prognosis and healthy controls. This was consistent with previous findings. Patients with active disease had significantly (*p* = 0.023) higher levels compared to patients in remission. Together, these findings suggest a higher level of MCP-1 in urine in active disease compared to when in remission. Chronic grumbling disease did not show significantly higher levels of uMCP-1 compared to remission.

Patients with kidney damage according to VDI had significantly higher uMCP-1 than patients without kidney damage. Patients with kidney damage had in higher extent severe prognosis, compared to patients without kidney damage. The patients with kidney damage correlated well with the patients with poor prognosis (data not shown), and it has been shown in previous studies that kidney damage correlate with a more severe overall outcome [15–17].

In the previous study, it was discussed why higher levels of uMCP-1 is associated with severe prognosis [21]. Two explanations are presented: either uMCP-1 signals constant subclinical disease activity and this ongoing inflammation is harmful over time, causing a more severe course of the disease, or uMCP-1 is a sign of renal damage which in turn is known to be associated with severe prognosis. MCP-1 is produced locally by kidney cells, and it not only recruits macrophages causing indirect damage but it also can directly start a fibrotic response in glomerular mesangial cells [41]. Our previous study showed a correlation with urinary protein HC and S-creatinine, and the correlation with S-creatinine was also seen in this study. These are indicators of renal damage, supporting the explanation of uMCP-1 as a marker of kidney damage. uMCP-1 has been shown to correlate with disease activity and with renal involvement in patients with SLE [24]. Furthermore, uMCP-1 is also associated with renal damage in diabetic nephropathy and it has been concluded that proteinuria increases the MCP-1 expression which accelerates diabetic nephropathy [42]. It is reasonable to believe that this is also the case in AAV and that MCP-1 is involved in the pathogenesis as well as being a biomarker of disease activity and prognosis.

Showing a high negative predictive value, low uMCP-1 values can to a high extent rule out a severe prognosis. This could be a help in adjusting the treatment more individually, perhaps with lower dosages or faster withdrawal of immunosuppressants. The positive predictive value was lower, but at the same time, we saw that patients with the highest values of uMCP-1 solely had severe prognosis (data not shown). This could be a guideline for more aggressive treatment. A proteomics study from 2009 defined a number of biomarkers that were able to distinguish between patients with active AAV and patients in remission. Proteolytic products of hemoglobin were the most frequently observed, but fragments of albumin and a1-antitrypsin were also seen. The study suggested the usefulness of a panel of urinary biomarkers [23]. uMCP-1 could be a good complement to other markers in such a panel. Continued research in the area is needed.

There are some studies on genetic correlations with AAV. In the 2012 European GWAS, AAV was associated not only with MHC but also with a single-nucleotide polymorphism (SNP) in the SERPINA1 locus. The strongest genetic associations were seen with the ANCA specificities; ANCA against PR3 was associated with HLA-DP and the genes encoding for *α*1-antitrypsine and proteinase 3. It was also shown that MPA and GPA are genetically distinct [43]. Here, we explored the association of SNPs in the CCR2 gene and the MCP-1 gene. There were no significant differences in the different genotypes in the CCR2 in the context of prognosis or uMCP-1 levels. The A/A genotype at -2518 in the MCP-1 gene was associated with severe prognosis and higher uMCP-1 levels than the A/G and G/G genotype. The SNP is situated in the regulatory region of MCP-1 and has been seen to increase the expression of MCP-1 in healthy individuals. The SNP has been shown to be associated with various diseases, like SLE [27, 44], systemic sclerosis [45], pulmonary tuberculosis [46], psoriasis [47], kidney transplant outcome [48], type 1 diabetes [49], renal disease progression in IgA nephritis [50], kidney failure in patients with diabetes mellitus type 2 [51], lupus nephritis [27], and severity in Behcet's disease [28]. The studies mentioned show somewhat confusing results, associating either the A allele or the G allele with risk of disease and increased MCP-1 expression. The cause of these divergent results is obscure. As mentioned before, our group demonstrated a deviant profile of cytokines in patients with AAV compared to healthy individuals [21, 52], and disease-specific conditions like this could play a role. The genetic differences between the populations in the different studies are another factor to consider. There are two meta-analyses from 2016. Lee and Bae gathered in total 14 publications and 3038 patients suffering from vasculitis, RA, or MS and found no significant association with the G allele [53]. On the other hand, Chen et al. associates the A allele with an increased risk of autoimmunity in their meta-analysis of studies on RA, Crohn's disease, and lupus nephritis [54]. The number of patients in the present study is rather small, and a larger study is needed to further investigate the association between MCP-1 polymorphism and AAV prognosis. There is also a need for further studies of the local production of MCP-1 in the kidney and migration of monocytes/macrophages to the renal tissue in relation to the MCP-1 -2518 A/G polymorphism.

## 5. Summary

In conclusion, MCP-1 levels in urine can be useful in determining if a patient is prone to have a more severe outcome. MCP-1 levels are significantly higher in patients with active disease and in patients with kidney damage. Polymorphism -2518A homozygous is associated with a more severe outcome and higher levels of uMCP-1, whereas CCR2-V64I did not show such a correlation. Further research is needed in the field.

## Figures and Tables

**Table 1:** Characteristics of patients included in the study. Patients with AAV followed at SUS, Lund, 2002–2011.

Number of patients	113
Number of urine samples	670
Urine samples per patient, median (range)	5 (1–22)
Proportion women	50.0% (*n* = 57)
Age at last follow-up, years (range)	62.6 (21–91)
Deceased at last follow-up	23
*Diagnosis*	
Diagnosis confirmed with renal PAD	62
GPA	74
MPA	39
*ANCA specificity*	
MPO	40
PR3	68
PR3 + MPO	1
Negative	3

AAV: antineutrophil cytoplasmic autoantibodies- (ANCA-) associated vasculitis; PAD: pathologic anatomic diagnosis; GPA: granulomatosis with polyangitis; MPA: microscopic polyangitis; MPO: myeloperoxidase; PR3: proteinase 3.

**Table 2:** Correlation between uMCP-1 and other biomarkers (^∗^*p* < 0.05, ^∗∗^*p* < 0.001).

		S-CRP	S-LPK	S-creatinine	S-cystatin C	Urine albumin creatinine index	Anti-PR3-ANCA	Capture PR3-ANCA	Anti-MPO-ANCA	BVAS
uMCP-1	Correlation coefficient	0.28^∗∗^	0.101	0.104	−0.09	0.24^∗∗^	0.005	0.10	0.09	0.16^∗∗^
	*p* value	0.0002	0.057	0.052	0.526	0.0003	0.952	0.204	0.477	0.0002
	*N*	348	352	351	54	247	134	180	64	629
uMCP-1/urine creatinine index	Correlation coefficient	0.29^∗∗^	0.16^∗^	0.19^∗∗^	−0.17	0.33^∗∗^	−0.03	0.14	0.14	0.15^∗∗^
	*p* value	0.0003	0.003	0.0004	0.224	0.0006	0.746	0.06	0.256	0.0002
	*N*	346	350	349	54	246	134	180	64	627

uMCP-1: urinary monocyte chemoattractant protein; S-CRP: serum C-reactive protein; LPK: white blood cell count; PR3: proteinase 3; ANCA: antineutrophil cytoplasmic autoantibodies; MPO: myeloperoxidase; BVAS: Birmingham vasculitis activity score.

**Table 3:** Distribution of genotypes of the -2518 MCP-1 and the CCR2-V64I polymorphism.

	Patients versus controls		ANCA specificity			Diagnosis		Prognosis	
	Patients	Healthy controls	MPO	PR3	Negative	GPA	MPA	Severe	Nonsevere
*MCP-1 -2518 A/G*									
A/A	54.5% (*n* = 30)	50% (*n* = 86)	63.2% (*n* = 12)	50.0% (*n* = 17)	0% (*n* = 0)	56.4% (*n* = 22)	46.7% (*n* = 7)	87.5% (*n* = 14)	39.5% (*n* = 15)
A/G	41.8% (*n* = 23)	50% (*n* = 86)	36.8% (*n* = 7)	47.1% (*n* = 16)	0% (*n* = 0)	38.5% (*n* = 15)	53.3% (*n* = 8)	12.5% (*n* = 2)	59.3% (*n* = 21)
G/G	3.6% (*n* = 2)	0% (*n* = 0)	0% (*n* = 0)	2.9% (*n* = 1)	100% (*n* = 1)	5.1% (*n* = 2)	0% (*n* = 0)	0% (*n* = 0)	5.3% (*n* = 2)
*CCR2* *V64I*									
G/G	84.2% (*n* = 48)	86.9% (*n* = 152)	80.0% (*n* = 16)	88.6% (*n* = 31)	100% (*n* = 1)	90.2% (*n* = 37)	73.3% (*n* = 11)	84.2% (*n* = 16)	86.5% (*n* = 32)
G/A	14.0% (*n* = 8)	12.0% (*n* = 21)	20.0% (*n* = 4)	8.5% (*n* = 3)	0% (*n* = 0)	7.3% (*n* = 3)	26.7% (*n* = 4)	15.8% (*n* = 3)	10.8% (*n* = 4)
A/A	1.8% (*n* = 1)	1.1% (*n* = 2)	0% (*n* = 0)	2.9% (*n* = 1)	0% (*n* = 0)	2.4% (*n* = 1)	0% (*n* = 0)	0% (*n* = 0)	(2.7% (*n* = 1)

MCP-1: monocyte chemoattractant protein-1; CCR2: CC chemokine receptor 2; MPO: myeloperoxidase; PR3: proteinase 3; GPA: granulomatosis polyangitis; MPA: microscopic polyangitis. Patients with severe prognosis had significantly higher frequency of the MCP-1 -2518 A/A genotype than patients with less severe prognosis (*p* < 0.05).
